# Case Report: Metagenomic next-generation sequencing applied in diagnosing psittacosis caused by *Chlamydia psittaci* infection

**DOI:** 10.3389/fcimb.2023.1249225

**Published:** 2023-09-20

**Authors:** Wan Xu, Qing Wang, Lin Li, Binghua Zhu, Qingqing Cai, Xiaoli Yi, Rong Fang, Qian Wang

**Affiliations:** ^1^Department of Emergency Internal Medicine, Shuguang Hospital Affiliated to Shanghai University of Traditional Chinese Medicine, Shanghai, China; ^2^Genoxor Medical Science and Technology Inc., Shanghai, China

**Keywords:** psittacosis, *Chlamydia psittaci*, infection, diagnosis, mNGS

## Abstract

**Background:**

*Chlamydia psittaci* is the causative agent of psittacosis in humans, while its rapid identification is hampered due to the lack of specificity of laboratory testing methods.

**Case presentation:**

This study reports four cases of *C. psittaci* infection after contact with a domestic parrot, all belonging to the same family. Common manifestations like fever, cough, headache, nausea, and hypodynamia appeared in the patients. Metagenomic next-generation sequencing (mNGS) aided the etiological diagnosis of psittacosis, revealing 58318 and 7 sequence reads corresponding to *C. psittaci* in two cases. The detected *C. psittaci* was typed as ST100001 in the Multilocus-sequence typing (MLST) system, a novel strain initially reported. Based on the results of pathogenic identification by mNGS, the four patients were individually, treated with different antibiotics, and discharged with favorable outcomes.

**Conclusion:**

In diagnosing psittacosis caused by a rare *C. psittaci* agent, mNGS provides rapid etiological identification, contributing to targeted antibiotic therapy and favorable outcomes. This study also reminds clinicians to raise awareness of psittacosis when encountering family members with a fever of unknown origin.

## Introduction

Psittacosis is a worldwide infectious disease caused by *Chlamydia psittaci* (*C. psittaci*), a zoonotic agent with a broad host range and complicated transmission ways (Tolba et al., 2019). The primary human infection route of *C. psittaci* is the inhalation of aerosols from contaminated animal excreta via the respiratory tract when in close contact with the infected bird species (Hogerwerf et al., 2017). After inhaled through the human lung, *C. psittaci* invades the bloodstream and proliferates in the liver, spleen, and mononuclear phagocyte systems, and spreads to the whole body through the bloodstream, affecting the lung, liver, spleen, kidney, and the central nervous system (Knittler and Sachse, 2015; Wang et al., 2021). Therefore, the clinical manifestations may vary from asymptomatic infection to severe atypical pneumonia or even fatal meningitis. The typical symptoms include chills, abrupt onset and remittent fever, headache, cough, myalgia, and hypodynamia (Wallensten et al., 2014; Chen et al., 2020). Due to the atypical clinical manifestations, rapid diagnosis of psittacosis is crucial for preventing severe illness.

So far, the identification of *C. psittaci* relies on several traditional means, including the isolation and identification of the pathogen, immunofluorescence method, indirect hemagglutination inhibition test, complement fixation test, enzyme-linked immunosorbent assay, the polymerase chain reaction and real-time polymerase chain reaction (Nieuwenhuizen et al., 2018). However, these detection approaches are all limited by either low sensitivity, high experimental requirement, or demand with a suspected pathogen, for which they are not suitable for the conventional detection of *C. psittaci*. For this reason, psittacosis is easily underdiagnosed and misdiagnosed, especially for clinicians who are unacquainted with this disease.

Metagenomic next-generation sequencing (mNGS) is a novel tool that rapidly and precisely identifies pathogenic microorganisms, regardless of bacteria, viruses, fungi, and parasites (Gu et al., 2019). With high throughput and low expense features, mNGS has been frequently applied for etiological diagnosis, specifically in the disease whose pathogen could not be recognized by traditional approaches, like *C. psittaci* (Duan et al., 2022). Herein, we describe a cluster of psittacosis cases in China, composed of four cases, aiming to highlight the contribution and superiority of mNGS in distinguishing this rare pathogen.

## Case presentation

**Figure 1:**
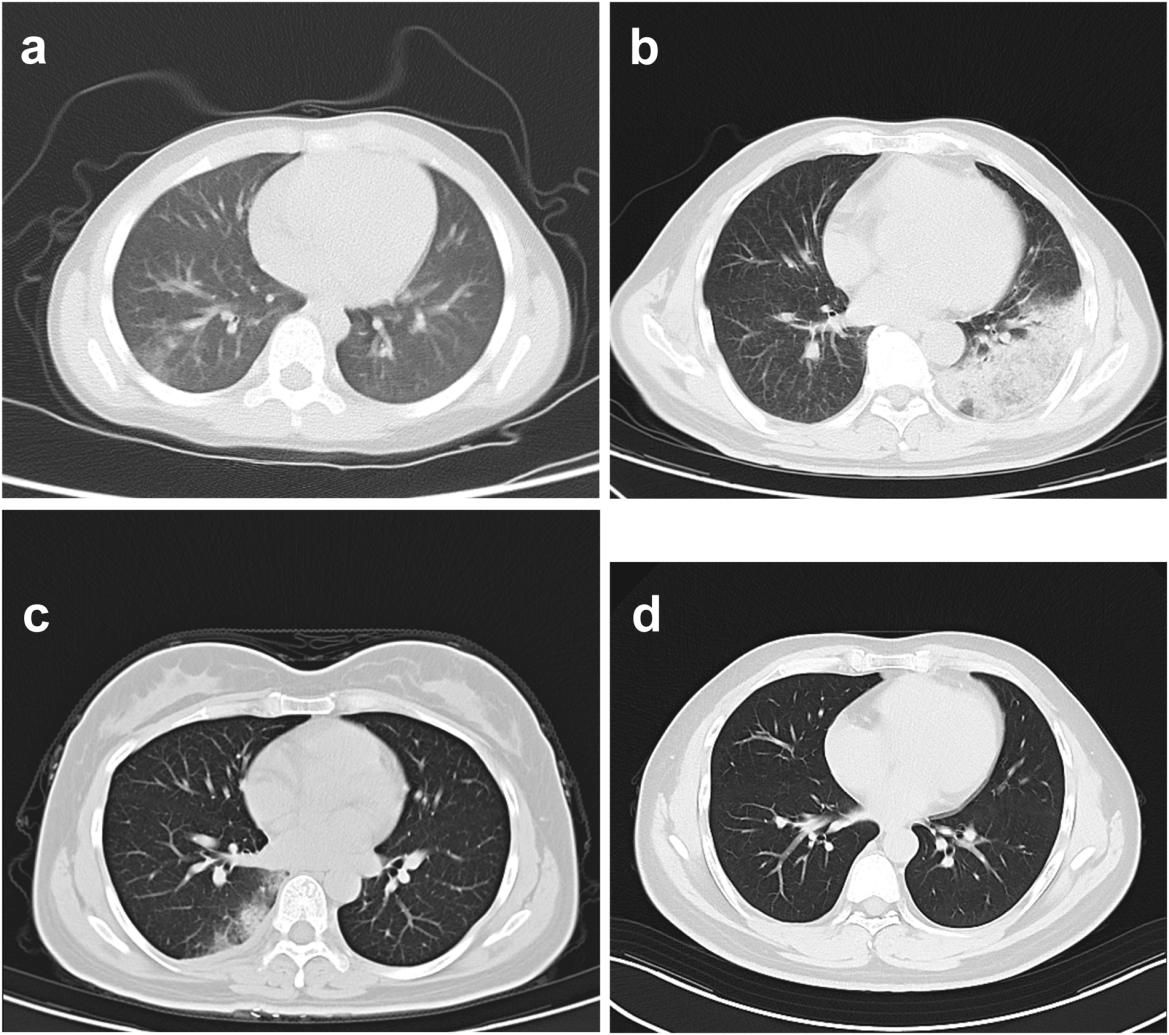
Chest CT indicates inflammation and other lesions in the lung of four patients. **(A)** Chest CT in case 1; **(B)** Chest CT in case 2; **(C)** Chest CT in case 3; **(D)** Chest CT in case 4.

Case 1: On 3 November 2022, a five years old girl (case 1) who resides in the urban area of Shanghai, east China, developed symptoms of nasal stuffiness and cough, with a body temperature of 37.3°C (**Table 1**). Then she took azithromycin suspension (1.5g qd po) for three days at home. Until 6 November 2022, the above symptoms were relieved, and her temperature returned to normal. Remitted cough appeared on 15 November 2022 in this patient, with less sputum, and she was admitted to our hospital (**Supplementary Table 1**). Chest computed tomography (CT) indicated inflammation in the upper and lower lobe of the right lung (**Figure 1A**). Interviews with the relatives regarding potential risk factors revealed that her family purchased a parrot in the bird and flower market on 24 October 2022, and the parrot died on 9 November 2022. On 15 November 2022, she took azithromycin (0.2g qd po), but no relief was noticed in the cough until the next day.

**Table 1:** Basic information and timeline of disease occurrence and development in the cases.

Items	Case 1	Case 2	Case 3	Case 4
Sex	Female	Male	Female	Male
Age (y)	5	57	35	34
Family relations with the initial case	Initial case	Grandfather	Mother	Father
Date of contact with the parrot	2022-10-24	2022-10-24	2022-10-24	2022-10-24
Date of symptom onset	2022-11-3	2022-11-13	2022-11-15	2022-11-18
Initial body temperature	37.3°C	39.5°C	39.8°C	37.2°C
Date of hospitalization	2022-11-19	2022-11-18	2022-11-19	–
Date of discharge	2022-11-24	2022-11-24	2022-11-24	–

On 19 November 2022, her cough continued, so she was hospitalized with a tentative diagnosis of community-acquired pneumonia (suspected *C. psittaci* infection) for further treatment. Blood routine results revealed abnormal proportions of lymphocytes (51.14%) and neutrophils (39.84%). High CD4+ T cell proportion (50.9%) and a high ratio of CD4 to CD8 (CD4/CD8, 2.06) were also observed (**Supplementary Table 1**). On the same day, respiratory pathogens, including the *Mycoplasma pneumoniae*, *Chlamydia pneumoniae*, *Adenoviridae*, *Respiratory syncytial virus*, *Influenza A virus*, and *Influenza B* virus, were detected using an indirect immunofluorescence test. The Influenza B virus antibody IgM test revealed a positive result: 67.84 (+), but no other respiratory pathogen was identified. Classical microbiological culture methods in blood and sputum for other typical bacteria were negative. No *mycobacterium tuberculosis* was detected. From 19 November 2022, the patient was treated with azithromycin (0.2g ivgtt qd) for three days in the hospital.

**Figure 2:**
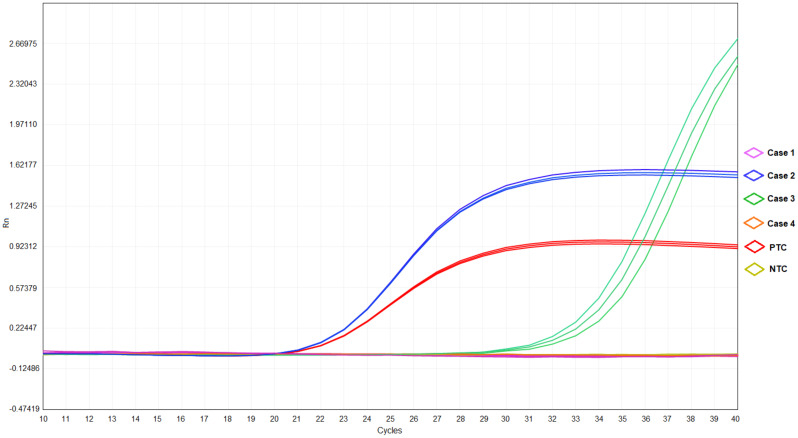
The results of qRT-PCR in cases 1-4, positive template control (PTC), and negative template control (NTC).

Throat swab-mNGS performed on 22 November 2022 identified sequences of *Haemophilus parainfluenzae* (242973 reads) and *Streptococcus pneumoniae* (7459 reads), but no *C. psittaci*-specific sequence was detected. The online **Supplementary Material** provided detailed information on sample collection and mNGS procedure (**Data Sheet 1**). The mNGS data generated in this study have been deposited in the NCBI Sequence Read Archive under BioProject accession number PRJNA951887. Ultimately, the final diagnosis of case 1 was community-acquired pneumonia (suspected psittacosis) accompanied by *influenza B virus* infection. After then, her cough symptom disappeared, and she was discharged On 24 November 2022. The timelines of contact, disease occurrence and development, and recovery are demonstrated in **Table 1**. Finally, quantitative real-time PCR (qRT-PCR) performed for *C. psittaci* detection in her throat swab sample revealed a negative result (**Table 2**; **Figure 2**). The online **Supplementary Material** included detailed information on qRT-PCR test primers, amplification mixture (**Supplementary Table 2**), and reaction procedure (**Supplementary Table 3**).

**Table 2:** The results of mNGS and qRT-PCR tests in the four cases.

Methods	Items	Case 1	Case 2	Case 3	Case 4
mNGS	Test date	2022-11-22	2022-11-19	2022-11-22	2022-11-23
	Types of sample	Throat swab	BALF	BALF	Sputum
	Results (Reads, relative abundance)	*S. pneumoniae* (7459, 0.45%)	*C. psittaci* (58318, 86.1%)	*C. psittaci* (7, 13.58%)	*S. pneumoniae* (12053, 1.79%)
qRT-PCR (*C. psittaci*)	Test date	2023-6-26	2023-6-26	2023-6-26	2023-6-26
	Types of sample	Throat swab	BALF	BALF	DNA
	Results	Negative	Positive	Positive	Negative

BALF, bronchoalveolar lavage fluid.

Case 2: This is case 1’s grandfather, a 57-year-old male, who lived in the same house in the urban area of Shanghai, China, as case 1. At noon on 13 November 2022, the patient felt nausea without apparent cause and experienced a fever (temperature of 39.5°C), nausea, and hypodynamia at night on the same day (**Supplementary Table 4**). Ibuprofen (0.2g qd po) and cephalosporin (0.2g bid po) were taken for three days while the symptoms were recurrent. He came to a local hospital on 13 November 2022 and was examined by chest CT, which revealed inflammation in the left lower lung (**Figure 1B**). Anti-infection therapy was adopted by administrating the second-generation cephalosporins (0.25g tid po) for two days. As the condition was not improved, he was admitted to our hospital and hospitalized on 18 November 2022 with a fever (temperature of 39°C), hypodynamia, and sore limbs. The admission examination demonstrated relatively high levels of white blood cells, neutrophil proportion, C-reaction protein, and procalcitonin, high CD4/CD8 (2.46), and significant signs of liver injury (**Supplementary Table 4**). No respiratory pathogen was identified. Classical microbiological culture methods in blood and sputum for other typical bacteria found none.

Epidemiological investigation indicated his contact history with the parrot, as in case 1. Consequently, this patient’s bronchoalveolar lavage fluid (BALF) sample was collected and detected by mNGS for etiological diagnosis on 19 November 2022. The online **Supplementary Material** provided detailed information on sample collection and mNGS procedures (**Data Sheet 1**). The result obtained on 20 November 2022 reported a reasonably high abundance of *C. psittaci* genome (58318 reads), with a relative abundance of 86.1% (**Table 2**) and a coverage rate of 97.20% (**Supplementary Figure 1**). The results of mNGS were confirmed by performing a qRT-PCR test for *C. psittaci*, in which a positive outcome was obtained (**Table 2**; **Figure 2**). The detected *C. psittaci* was typed as ST100001 in the Multilocus-sequence typing (MLST) system, a new MLST profile (**Supplementary Table 5**). The patient was diagnosed with community-acquired pneumonia (*C. psittaci* infection), hyponatremia, and liver injury and symptomatically treated with piperacillin sodium/tazobactam sodium for injection (4.5g q12h) (day 1-7) combined azithromycin (0.5g ivgtt qd) (day 1) or moxifloxacin (0.4g ivgtt qd) (day 2-7), and glutathione combined magnesium isoglycyrrhizinate injection (day 1-7) for liver protection. Six days later (24 November 2022), he was discharged after returning close to a premorbid condition.

Case 3: This is a 35-year-old female who is case 1’s mother and lives with the family members. They lived together as a family. In the afternoon of 15 November 2022, she got a fever, and the temperature reached 39.8°C, accompanied by headache, muscular soreness, rigor, hypodynamia, chest stuffiness, and anhelation. No improvement was found after oral administration of Lianhua Qingwen capsule (4 capsules tid) and benorilate tablets (0.5g qd po), so she was admitted to the local hospital on 16 November 2022 (**Supplementary Table 6**). Chest CT demonstrated inflammation accompanied by partial pleural thickening at the lower lobe of the right lung and calcification at the upper lobe of the right lung (**Figure 1C**). Her symptoms eased after taking the second-generation cephalosporins (0.25g tid po). However, the fever, headache, hypodynamia, and muscular stiffness recurred on 18 November 2022, which was not improved after administration of Minocycline hydrochloride capsules (200mg qd po).

On 19 November 2022, she was received as an inpatient in our hospital, tentatively diagnosed with community-acquired pneumonia in the emergency department. Her contact history with the parrot was the same with case 1. Blood routine showed a 15.11% proportion in monocytes. Abnormalities in 56.79mg/L of C-reaction protein level and 3.53mmol/L of K ion concentration were noted. No respiratory pathogen was identified. Classical microbiological culture methods in blood and sputum for other typical bacteria were negative. No *mycobacterium tuberculosis* was detected. After admission to the hospital, she was treated with moxifloxacin (0.4g ivgtt qd) (day 1) and the fever was kept down on the next day, then the drug was changed into doxycycline (0.1g q12h po) (day 2-5). BALF-mNGS test was conducted in case 3 on 22 November 2022, and *C. psittaci* (7 reads, 13.58% of relative abundance) was also identified on 24 November. A positive result for *C. psittaci* was also generated by the qRT-PCR test in her BALF sample (**Table 2** and **Figure 2**). Therefore, the final diagnosis was community-acquired pneumonia (*C. psittaci* infection), and the above treatment was maintained. On 24 November 2022, she was discharged in good condition.

Case 4: This patient is case 1’s father, a 34-year-old male, who lives as a family member with the above cases. On 18 November 2022, he developed symptoms of headache and hypodynamia, with a self-detected temperature of 37.2°C (**Supplementary Table 7**). Blood routine in the emergency department on the same day showed no abnormality. Chest CT showed a slight fibrosis at the middle lobe of the right lung and a minor thickening at the lower bilateral pleura (**Figure 1D**). From 18 November 2022 to 20 November, minocycline capsules (200mg qd po) were taken. On 21 November 2022, this patient was further examined in blood routine (43% of neutrophils and 46.8% of lymphocyte) and chest CT (no difference was found with the last examination). Doxycycline (0.1g q12h po) replaced minocycline in the following three days, and his symptoms disappeared. On 23 November 2022, sputum-mNGS was performed, but no *C. psittaci* was detected. No *C. psittaci* was detected by qRT-PCR test (**Table 2** and **Figure 2**).

## Discussion

In this case report, infection by *C. psittaci* was presented, whose etiologic diagnosis was aided by mNGS and supported by the favorable outcome after targeted treatment. The whole process of this event, including the route of infection, the symptoms and diagnosis of disease, and the personalized treatment of disease, is distinct, which offers a reliable reference for clinicians when facing a potential psittacosis episode.

Avian species are the natural host for *C. psittaci* (Johnson and Grimes, 1983). Still, other animals, including humans, can get infected after contact with birds by inhaling dried contaminated bird secretions, dried-out droppings, or dust from feathers (Hogerwerf et al., 2017). In the present study, the parrot was probably to get infected before being purchased from the bird and flower market by the family (on 24 October 2022) and died from the *C. psittaci* infection (on 9 November 2022), with a duration of 17 days. In the family’s community in the urban area of Shanghai, east China, nothing is unusual in the environment, ecology, urbanization, and pet habits. A recent report reveals that *C. psittaci* pneumonia was identified in 9 patients from November 2018 to December 2021 at Huashan Hospital in Shanghai (Zhu et al., 2023), suggesting a relatively low prevalence of psittacosis in this region. From the perspective of the chronological order of symptoms appearance, the initial case (case 1) was more likely infected by the contagious parrot, with a reasonable incubation period of 11 days (from 24 October 2022 to 3 November 2022). The contact history with parrots is a vital epidemiological evidence for raising awareness and establishing the diagnosis and treatment procedure pointing to psittacosis (Chaber et al., 2021). It is a pity that the parrot was not tested because it had died and been discarded before the second case was symptomatic. This study implies a low awareness of psittacosis in the general population, even in bird breeders.

However, the infection route of the other three cases (cases 2-4) is uncertain, direct from the infected parrot or by human-to-human transmission through case 1. Report on the human-to-human transmission of *C. psittaci* is rare. In 2013, multiple human-to-human transmission from a severe case of psittacosis in Sweden was described; one patient severely ill transmitted the disease to two family members, one hospital roommate, and seven hospital caregivers (Wallensten et al., 2014). In China, the first documented report of human-to-human transmission of *C. psittaci* was from a hospital in Shangdong province, in which most of the initial patients worked at a duck-meat processing plant (Zhang et al., 2022). But in a cluster of psittacosis cases in which all the patients lived together, as in the present study, further investigation and sufficient evidence were needed to estimate whether it is a human-to-human transmission of psittacosis. Meanwhile, clinicians should raise alertness of psittacosis when encountering family members with a fever of unknown origin, thereby realizing the early detection of this rare agent.

As Tolba et al. reported, *C. psittaci*-positive cases were closely associated with older persons (≥30 years) who had respiratory signs and handled birds in pet markets (Tolba et al., 2019). In a cluster of psittacosis cases in Zhejiang Province of China, the patients range from 42-70 years old (Yao et al., 2022). Nevertheless, we are reporting a highly suspected younger case of *C. psittaci*-infection (case 1, five years old), which is extremely rare. It may be attributed to her co-infection with the *influenza B virus*. Co-infections of the influenza virus and bacteria in the respiratory tract have been proven to present respiratory manifestations (Mancini et al., 2005; Mancini et al., 2008). Meanwhile, earlier onset of symptoms was observed in the child (case 1) and older (case 2) individuals (**Table 1**), with higher CD4+ T cell proportion (50.9%) and CD4/CD8 (2.06) in case 1 (**Supplementary Table 1**) and higher CD4/CD8 (2.46) in case 2 (**Supplementary Table 2**) indicated abnormal immune status. It has been proven that virulent *C. psittaci* infection suppresses immune responses by inhibiting humoral responses, increasing mortality in H9N2-infected birds (Chu et al., 2016). Moreover, T lymphocytes, especially CD8-positive cells, play a significant role in the cellular immune response against *C. psittaci* (Niemczuk and Bednarek, 2003). Hence, the current cases report, containing diverse age groups, suggests a trend that populations with lower immunity are prone to be infected with *C. psittaci*, with earlier onset of the disease and more severe clinical symptoms.

Due to the variable clinical presentations, absence of epidemiological information, and low clinicians’ awareness of this disease, rapid diagnosis of psittacosis is still a challenge in clinical, and delayed use of effective antibiotics may lead to high mortality in severe cases. For rare pathogens such as *C. psittaci*, mNGS has been recommended and applied for etiologic diagnosis, which significantly benefits the diagnosis of severe pneumonia (Chen et al., 2020; Wu et al., 2021; Tang et al., 2022). In our patients, an accurate diagnosis of psittacosis mainly relies on an overall consideration of clinical characteristics, a specific epidemiological history, and an mNGS test for etiologic identification. Common manifestations like cough, nausea, sudden fever, headache, myalgia, rigor, and hypodynamia are presented in the patients. A contact history with the parrot was confirmed. Recently, a family outbreak of psittacosis was reported in China, in which two newly purchased pet parrots who had died were probably the primary source of infection (Li et al., 2021). In this study, the patients were treated with anti-infective therapy based on suspicion before mNGS detection, but lacked adequate response. Therefore the antibiotics were adjusted (from azithromycin to moxifloxacin in case 2 and moxifloxacin to doxycycline in case 3) after mNGS reporting, and favorable outcomes were observed in them, reversely supporting the diagnosis of *C. psittaci* infection. Ultimately, the sequence data of *C. psittaci* was blasted for typing in the MLST system, a”gold standard” methodology for molecular characterization of strains in *Chlamydiales* barcoding them as sequence types (STs) (Anstey et al., 2021). The *C. psittaci* identified in this study was typed as ST100001, a new MLST profile reported initially. Thus, its infectivity and pathogenicity remain to be explored.

To summarize, we reported an episode of cluster infection caused by *C. psittaci*, with complete and precise procedures on infection route, diagnosis, and treatment, highlighting the contribution of mNGS in etiological diagnosis. The limitation of this case report is that *C. psittaci* was not detected in cases 1 and 4 by mNGS and qRT-PCR, probably because of a low load of the pathogen in the mild-symptom instances, different types of samples used for mNGS, or the usage of antibiotics for a long time. But their contact history with the bird and the family members, typical symptoms, and the effective response to the drugs specific for psittacosis are all strong evidences of infection with *C. psittaci*. To sum up, effective prevention and control measures are appealed to prevent the spread of *C. psittaci* among psittacine birds and humans in close contact with birds.

## Data availability statement

The datasets presented in this study can be found in online repositories. The names of the repository/repositories and accession number(s) can be found below: [https://www.ncbi.nlm.nih.gov/bioproject/ANDPRJNA951887].

## Ethics statement

This study involving human participants have been approved by the ethics committee of Shuguang Hospital Affiliated to Shanghai University of Traditional Chinese Medicine. Written informed consent for publication of the individual person’s data (individual details and images) was obtained from the patients and the patients’ legal guardian. Written informed consent was obtained from the participant/patient(s) for the publication of this case report.

## Author contributions

QiaW was involved in managing the patients, and she had the idea to submit the case report to the journal. RF engaged in the diagnosis process of the patients and their treatment. WX analyzed and interpreted the clinical data and wrote the article’s first draft. QinW contributed to the individual details collection and clinical data acquisition and was a major contributor in writing the manuscript. LL collected the samples for pathogen detection and arranged the relevant matters on physical examination. BZ performed the literature search and review and revised the previous version of the manuscript. QC and XY contributed to the whole process of mNGS detection, including sample reception, treatment, sequencing, data analysis, and report interpretation. All authors read and approved the final manuscript. All authors contributed to the article.

## Supplementary material

Supplementary Figure 1 The genome coverage rate of *C. psittaci* in case 2.

## References

[B1] Anstey S. I. Kasimov V. Jenkins C. Legione A. Devlin J. Amery-Gale J.. (2021). Chlamydia psittaci st24: Clonal strains of one health importance dominate in Australian horse, bird and human infections. Pathogens 10 (8), 1015. doi: 10.3390/pathogens10081015 34451478 PMC8401489

[B2] Chaber A. L. Jelocnik M. Woolford L. (2021). Undiagnosed cases of human pneumonia following exposure to chlamydia psittaci from an infected rosella parrot. Pathogens 10 (8), 968. doi: 10.3390/pathogens10080968 34451432 PMC8399200

[B3] Chen X. Cao K. Wei Y. Qian Y. Liang J. Dong D.. (2020). Metagenomic next-generation sequencing in the diagnosis of severe pneumonias caused by chlamydia psittaci. Infection 48 (4), 535–542. doi: 10.1007/s15010-020-01429-0 32314307 PMC7223968

[B4] Chu J. Zhang Q. Zhang T. Han E. Zhao P. Khan A.. (2016). Chlamydia psittaci infection increases mortality of avian influenza virus H9n2 by suppressing host immune response. Sci. Rep. 6, 29421. doi: 10.1038/srep29421 27405059 PMC4941526

[B5] Duan Z. Gao Y. Liu B. Sun B. Li S. Wang C.. (2022). The application value of metagenomic and whole-genome capture next-generation sequencing in the diagnosis and epidemiological analysis of psittacosis. Front. Cell Infect. Microbiol. 12. doi: 10.3389/fcimb.2022.872899 PMC9207344 35734579

[B6] Gu W. Miller S. Chiu C. Y. (2019). Clinical metagenomic next-generation sequencing for pathogen detection. Annu. Rev. Pathol. 14, 319–338. doi: 10.1146/annurev-pathmechdis-012418-012751 30355154 PMC6345613

[B7] Hogerwerf L. DEG B. Baan B. Van Der Hoek W. (2017). Chlamydia psittaci (Psittacosis) as a cause of community-acquired pneumonia: A systematic review and meta-analysis. Epidemiol. Infect. 145 (15), 3096–3105. doi: 10.1017/S0950268817002060 28946931 PMC9148753

[B8] Johnson M. C. Grimes J. E. (1983). Resistance of wild birds to infection by chlamydia psittaci of mamMalian origin. J. Infect. Dis. 147 (1), 162. doi: 10.1093/infdis/147.1.162 6822754

[B9] Knittler M. R. Sachse K. (2015). Chlamydia psittaci: Update on an underestimated zoonotic agent. Pathog. Dis. 73 (1), 1–15. doi: 10.1093/femspd/ftu007 25853998

[B10] Li N. Li S. Tan W. Wang H. Xu H. Wang D. (2021). Metagenomic next-generation sequencing in the family outbreak of psittacosis: The first reported family outbreak of psittacosis in China under covid-19. Emerg. Microbes Infect. 10 (1), 1418–1428. doi: 10.1080/22221751.2021.1948358 34176434 PMC8284143

[B11] Mancini D. A. Alves R. C. Mendonca R. M. Bellei N. J. Carraro E. MaChado A. M.. (2008). Influenza virus and proteolytic bacteria co-infection in respiratory tract from individuals presenting respiratory manifestations. Rev. Inst Med. Trop. Sao Paulo 50 (1), 41–46. doi: 10.1590/s0036-46652008000100009 18327486

[B12] Mancini D. A. Mendonca R. M. Dias A. L. Mendonca R. Z. Pinto J. R. (2005). Co-infection between influenza virus and flagellated bacteria. Rev. Inst Med. Trop. Sao Paulo 47 (5), 275–280. doi: 10.1590/s0036-46652005000500007 16302111

[B13] Niemczuk K. Bednarek D. (2003). Changes in the peripheral leukocyte phenotype of calves in clinical cases of bronchopneumonia complicated with chlamydial co-infectious agent. Pol. J. Vet. Sci. 6 (2), 125–129. 12817783

[B14] Nieuwenhuizen A. A. Dijkstra F. Notermans D. W. van der Hoek W. (2018). Laboratory methods for case finding in human psittacosis outbreaks: A systematic review. BMC Infect. Dis. 18 (1), 442. doi: 10.1186/s12879-018-3317-0 30165831 PMC6118005

[B15] Tang J. Tan W. Luo L. Xu H. Li N. (2022). Application of metagenomic next-generation sequencing in the diagnosis of pneumonia caused by chlamydia psittaci. Microbiol. Spectr. 10 (4), e0238421. doi: 10.1128/spectrum.02384-21 35938720 PMC9431268

[B16] Tolba H. M. N. Abou Elez R. M. M. Elsohaby I. (2019). Risk factors associated with chlamydia psittaci infections in psittacine birds and bird handlers. J. Appl. Microbiol. 126 (2), 402–410. doi: 10.1111/jam.14136 30353983

[B17] Wallensten A. Fredlund H. Runehagen A. (2014). Multiple human-to-human transmission from a severe case of psittacosis, Sweden, January-February 2013. Euro Surveill 19 (42), 20937. doi: 10.2807/1560-7917.es2014.19.42.20937 25358043

[B18] Wang L. Shi Z. Chen W. Du X. Zhan L. (2021). Extracorporeal membrane oxygenation in severe acute respiratory distress syndrome caused by chlamydia psittaci: A case report and review of the literature. Front. Med. (Lausanne) 8. doi: 10.3389/fmed.2021.731047 PMC8554049 34722571

[B19] Wu H. H. Feng L. F. Fang S. Y. (2021). Application of metagenomic next-generation sequencing in the diagnosis of severe pneumonia caused by chlamydia psittaci. BMC Pulm Med. 21 (1), 300. doi: 10.1186/s12890-021-01673-6 34556069 PMC8461849

[B20] Yao W. Chen X. Wu Z. Wang L. Shi G. Yang Z.. (2022). A cluster of psittacosis cases in lishui, Zhejiang Province, China, in 2021. Front. Cell Infect. Microbiol. 12. doi: 10.3389/fcimb.2022.1044984 PMC9798449 36590592

[B21] Zhang Z. Zhou H. Cao H. Ji J. Zhang R. Li W.. (2022). Human-to-human transmission of chlamydia psittaci in China, 2020: An epidemiological and aetiological investigation. Lancet Microbe 3 (7), e512–ee20. doi: 10.1016/S2666-5247(22)00064-7 35617977

[B22] Zhu N. Zhou D. Yuan R. Ruzetuoheti Y. Li J. Zhang X.. (2023). Identification and comparison of chlamydia psittaci, legionella and mycoplasma pneumonia infection. Clin. Respir. J. 17 (5), 384–393. doi: 10.1111/crj.13603 36929690 PMC10214566

