# Supplementary material for: Case Report: Metagenomic next-generation sequencing applied in diagnosing psittacosis caused by Chlamydia psittaci infection
Source: Front Cell Infect Microbiol. 2023 Sep 20;13:1249225. doi: 10.3389/fcimb.2023.1249225 (PMC10548267; doi:10.3389/fcimb.2023.1249225)
Supplement: Supplementary file 1 [file DataSheet_1.docx]

**Materials and methods**

**Sample collection**

Four specimens, including two bronchoalveolar lavage fluid (BALF) samples, one throat swab, and one sputum sample, were collected from four patients following the aseptic processing standards. Sampling was completed in the Shuguang Hospital Affiliated to Shanghai University of Traditional Chinese Medicine, where the patients were treated. The samples were stored in a sterile container and sent for pathogen detection by preserving them in dry ice.

**Metagenomic next-generation sequencing (mNGS)**

The whole mNGS process was conducted in the Genoxor Medical Science and Technology Inc. laboratory (Shanghai, China). After pre-treatment, the samples were subjected to DNA extraction with a TIANamp Micro DNA Kit (TIANGEN BIOTECH, Beijing, China) following the manufacturer’s operational manual. The total mass of extracted DNA was quantified using a QuantiT dsDNA HS Assay Kit and Qubit 3.0 Fluorometer (Thermo Fisher Scientific, USA). A total of 100ng of extracted DNA were subjected to processes of interruption, end repair, library construction, and sequencing. Agilent 2100 Bioanalyzer (Agilent Technologies, USA) was used for quality control. Sequencing was carried out in a single-end 75-bp mode using the NextSeq 500 system with 75 cycles Reagent Kit on an Illumina NextSeq 550 Dx sequencer.

**Bioinformatics analysis of species-level abundance profiling**

The short and low-quality reads in the raw data were filtered out by trimmomatic v0.36 (1). Then, the reads mapping to human reference genome GRCh37 were removed by using the short-read alignment tool Bowtie v2.2.6 (2). The remaining sequence data (archived in the NCBI's Sequence Read Archive with the accession number PRJNA951887) were aligned to the Microbial Genome Databases consisting of bacteria, viruses, fungi, and parasites. Only the mapped sequences were processed for advanced data analysis to produce the suspected pathogenic microorganisms lists, including the number of reads and coverage rate. Clinical diagnosis was made by considering all the clinical manifestations and suspected pathogens identified by mNGS and other laboratory tests.

**Quantitative** **real-time PCR (qRT-PCR)**

The extracted DNA from the above four samples was subjected to the quantitative qRT-PCR method using *C. psittaci*-specific primers (sequences: Forward-CACTATGTGGGAAGGTGCTTCA, Reverse-CTGCGCGGATGCTAATGG) in a SLAN96S thermal cycler (HONGSHI, Shanghai, China). Forty cycles were applied in the procedure of amplification. The amplification mixture is presented in Supplementary Table 2 and the reaction procedure is provided in Supplementary Table 3.

Supplementary Table 1 Timeline of physical examinations, diagnosis, and treatment in case 1

| Dates | Physical examination | Diagnosis and treatment |
| --- | --- | --- |
| 2022-11-3 | T: 37.3℃ | Azithromycin suspension (1.5g qd po) for three days |
| 2022-11-15 | Chest CT: inflammation in the upper and lower lobe of right lung  Lymphocytes: 51.14%  Neutrophils: 39.84% | Azithromycin (0.2g qd po) |
| 2022-11-16 |  | Azithromycin (0.2g ivgtt qd), ambroxol (2.5ml, tid) for three days |
| 2022-11-18 | CRP: <0.5mg/L |  |
| 2022-11-19 | Lymphocytes: 53.8%  Neutrophils: 38.5%  *Influenza B virus* antibody IgM: 67.84 (+)  Blood culture: negative  Sputum culture: negative  CD4+ T cell: 50.9%  CD4/CD8: 2.06 | Tentative diagnosis: community-acquired pneumonia (suspected *C. psittaci* infection)  Treatment: azithromycin (0.2g ivgtt qd) for three days |
| 2022-11-22 | PCT: 0.61 ng/mL  IL-6: 9.92 pg/mL | Final diagnosis: community-acquired pneumonia (suspected psittacosis), *influenza B virus* infection |

CT: Computed tomography; IgM: immunoglobulin M.

Supplementary Table 2 Amplification mixture of qRT-PCR

| Reagents | Volume (μl) |
| --- | --- |
| Hieff^®^ qPCR SYBR Green Master | 10 |
| Forward Primer (10μM) | 0.4 |
| Reverse Primer (10μM) | 0.4 |
| DNA template | 2 |
| Double-distilled water | 7.2 |
| Total volume (μl) | 20 |

Supplementary Table 3 The reaction procedure of qRT-PCR

| Steps | Temperature（°C） | Duration | Cycles |
| --- | --- | --- | --- |
| Pre-degeneration | 95 | 5min |  |
| Degeneration | 95 | 10s | 40 |
| Annealing/extending | 60 | 30s |  |
| Melting-curve stage | 72 | 5min |  |

Supplementary Table 4 Timeline of physical examinations, diagnosis, and treatment in case 2

| Dates | Physical examination | Diagnosis and treatment |
| --- | --- | --- |
| 2022-11-13 | Fever, T: 39.5℃ | Ibuprofen (0.2g qd po) and cephalosporin (0.2g bid po) for three days |
| 2022-11-16 | Chest CT: inflammation in the left lower lung | Second-generation cephalosporins (0.25g tid po) for two days |
| 2022-11-18 | Fever, T: 39.0℃  WBC: 7.07ⅹ10^9^/L  Neutrophils: 79%  CD4/CD8: 2.46  CRP: 357.18 mg/L  PCT: 1.7 ng/mL  TNI: 0.384  TBIL: 26.6 umol/L  ALT: 106 U/L  AST: 107 U/L  GGT: 244 U/L  AKP: 166 U/L  ALB: 30.5 g/L  Na: 127 mmol/L  K: 3.16 mmol/L  PT: 14.3 second  D-Dimer: 0.8 mg/L  Chlamydia pneumoniae IgG: 32.2 | Tentative diagnosis: community-acquired pneumonia, non-severe |
| 2022-11-19 | Blood culture: negative  Sputum culture: negative | Final diagnosis: community-acquired pneumonia (*C. psittaci* infection), hyponatremia, liver injury  Antibiotic treatment: piperacillin  sodium/tazobactam sodium for injection (4.5g q12h) combined azithromycin (0.5g ivgtt qd) (day 1) or moxifloxacin (0.4g ivgtt qd) (day 2-7); glutathione combined magnesium isoglycyrrhizinate injection (day 1-7) for liver protection |

WBC: white blood cell; CRP: C-reactive protein; PCT: procalcitonin; TNI: Troponin I; TBIL: total bilirubin; ALT: alanine aminotransferase; AST: aspartate aminotransferase; GGT: gamma-glutamyl transpeptidase; AKP: alkaline phosphatase; ALB: albumin; PT: prothrombin time; BALF: bronchoalveolar lavage fluid.

Supplementary Table 5 New MLST profiles found in *C. psittaci* from BALF sample of case 2

| ST | enoA | fumC | gatA | gidA | hemN | hflX | oppA | Hits |
| --- | --- | --- | --- | --- | --- | --- | --- | --- |
| 100001 | 13 | 100001 | 100001 | 13 | 9 | 11 | 13 | 1 |

Supplementary Table 6 Timeline of physical examinations, diagnosis, and treatment in case 3

| Dates | Physical examination | Diagnosis and treatment |
| --- | --- | --- |
| 2022-11-15 | Fever, T: 39.8℃ | Lianhua Qingwen capsule (4 capsules tid) and benorilate tablets (0.5g qd po) |
| 2022-11-16 | Chest CT: inflammation accompanied by partial pleural thickening at the lower lobe of the right lung, calcification at the upper lobe of the right lung | Second-generation cephalosporins (0.25g tid po) for two days |
| 2022-11-18 | Fever | Minocycline hydrochloride capsules (200mg qd po) |
| 2022-11-19 | Fever, T: 38.9℃  Monocytes: 15.11%  CD8+ T cell: 38.1%  CRP: 56.79 mg/L  PCT: 0.143 ng/mL  PT: 13.1 second  Fibrinogen: 5.82 g/L  D-Dimer: 0.76 mg/L  K: 3.53 mmol/L | Tentative diagnosis: community-acquired pneumonia (non-severe)  Treatment: moxifloxacin (0.4g ivgtt qd) (day 1), doxycycline (0.1g q12h po) (day 2-5) |
| 2022-11-22 | Blood culture: negative  Sputum culture: negative | Final diagnosis:community-acquired pneumonia (*C. psittaci* infection) |

Supplementary Table 7 Timeline of physical examinations, diagnosis, and treatment in case 4

| Dates | Physical examination | Diagnosis and treatment |
| --- | --- | --- |
| 2022-11-18 | T: 37.2℃  Chest CT: slight fibrosis at the middle lobe of the right lung, minor thickening at the lower bilateral pleura | Minocycline capsules (200mg qd po) for three days |
| 2022-11-21 | Neutrophils: 43%  Lymphocyte: 46.8%  Chest CT: without any change | Doxycycline (0.1g q12h po) for three days |
| 2022-11-23 |  | Diagnosis: upper respiratory infection (suspected psittacosis) |

**References**

1. Bolger AM, Lohse M, Usadel B. Trimmomatic: A Flexible Trimmer for Illumina Sequence Data. *Bioinformatics* (2014) 30(15):2114-20. doi: 10.1093/bioinformatics/btu170.

2. Langmead B, Salzberg SL. Fast Gapped-Read Alignment with Bowtie 2. *Nat Methods* (2012) 9(4):357-9. doi: 10.1038/nmeth.1923.
