# Supplementary figures and images for: Case Report: Metagenomic next-generation sequencing applied in diagnosing psittacosis caused by Chlamydia psittaci infection
Source: Front Cell Infect Microbiol. 2023 Sep 20;13:1249225. doi: 10.3389/fcimb.2023.1249225 (PMC10548267; doi:10.3389/fcimb.2023.1249225)

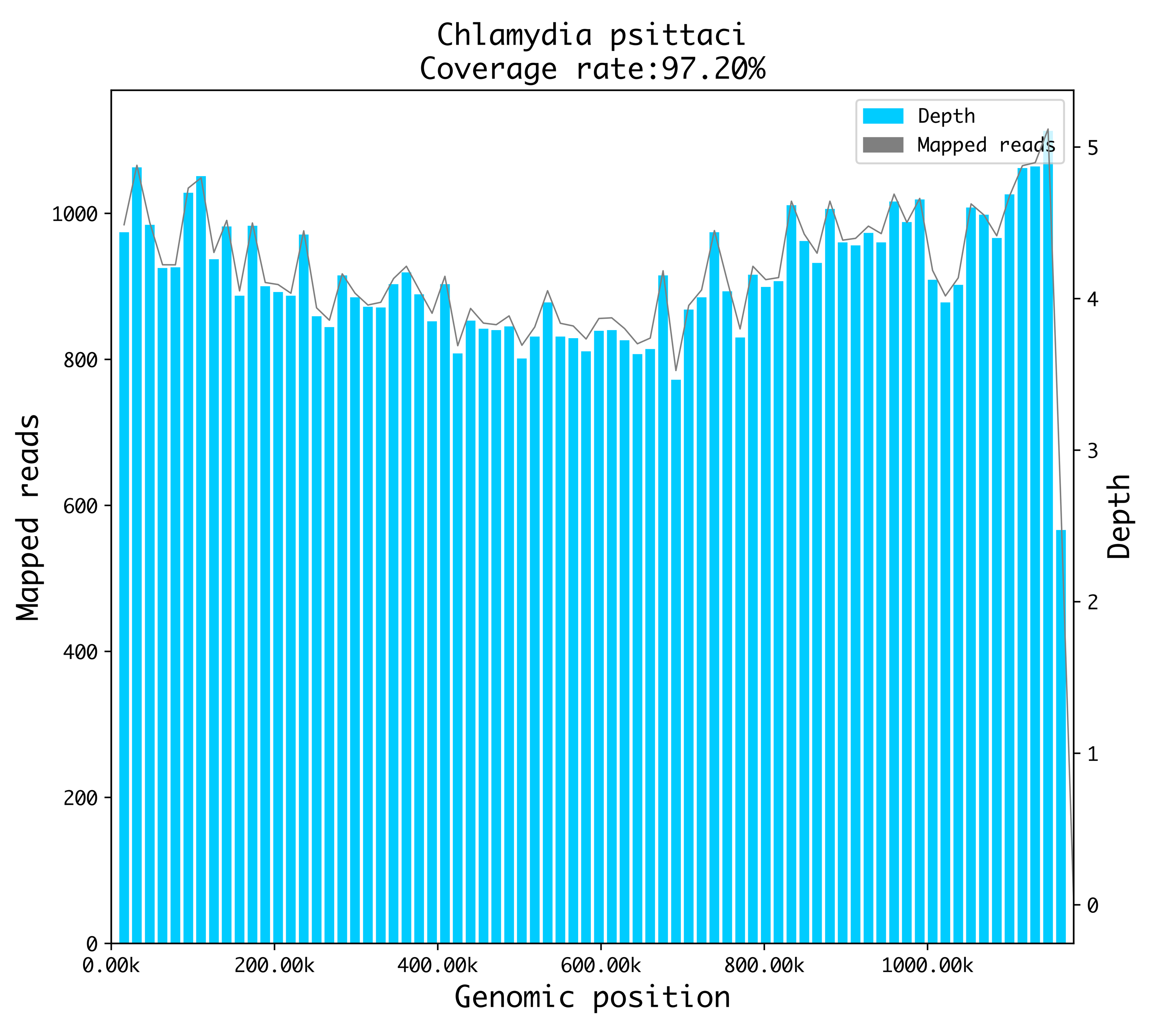

Supplement: Supplementary Figure 1 — The genome coverage rate of C. psittaci in case 2. [file Image_1.tif]
